# 4^th^ generation synchrotron source boosts crystalline imaging at the nanoscale

**DOI:** 10.1038/s41377-022-00758-z

**Published:** 2022-03-25

**Authors:** Peng Li, Marc Allain, Tilman A. Grünewald, Marcus Rommel, Andrea Campos, Dina Carbone, Virginie Chamard

**Affiliations:** 1 Aix-Marseille Univ, CNRS, Centrale Marseille, Institut Fresnel, Marseille, France; 2 Nanofabrication Laboratory, Department of Microtechnology and Nanoscience, MC2, Chalmers University of Technology, SE-412 96 Gothenburg, Sweden; 3 Aix Marseille Univ, CNRS, Centrale Marseille, FSCM (FR1739), CP2M, 13397 Marseille, France; 4 MAX IV Laboratory, Fotongatan 2, 225 94 Lund, Sweden; 5 Present Address: Diamond Light Source, Harwell Science and Innovation Campus, Fermi Ave, Didcot, OX11 0DE UK

**Keywords:** Imaging and sensing, X-rays, Microscopy

## Abstract

New 4^th^-generation synchrotron sources, with their increased brilliance, promise to greatly improve the performances of coherent X-ray microscopy. This perspective is of major interest for crystal microscopy, which aims at revealing the 3D crystalline structure of matter at the nanoscale, an approach strongly limited by the available coherent flux. Our results, based on Bragg ptychography experiments performed at the first 4^th^-generation synchrotron source, demonstrate the possibility of retrieving a high-quality image of the crystalline sample, with unprecedented quality. Importantly, the larger available coherent flux produces datasets with enough information to overcome experimental limitations, such as strongly deteriorated scanning conditions. We show this achievement would not be possible with 3^rd^-generation sources, a limit that has inhibited the development of this otherwise powerful microscopy method, so far. Hence, the advent of next-generation synchrotron sources not only makes Bragg ptychography suitable for high throughput studies but also strongly relaxes the associated experimental constraints, making it compatible with a wider range of experimental set-ups at the new synchrotrons.

## Abstract (web summary)

An unprecedented quality image is obtained at the first 4th generation synchrotron, which allows now compensating some experimental limitations.

## Graphical abstract

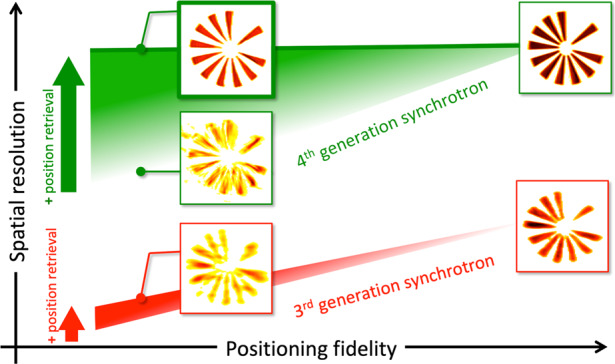

## Introduction

By squeezing an electron beam into extremely compact bunches, 4^th^-generation synchrotron sources produce highly brilliant X-ray beams and are expected to bring science to new frontiers^1^. The Max IV Laboratory in Sweden has pioneered the field with the installation of a multi-bend achromat-based storage ring, producing its first X-ray beam in 2015^2^, shortly followed by SIRIUS^3^ and ESRF-EBS in 2020^4^. These have paved the way for about ten more 4^th^-generation sources currently under construction or planned worldwide. The improvement of the magnetic lattice structure at the core of these new storage ring facilities led to a dramatic decrease in the electron beam transverse size and divergence. This in turn generates a large enhancement of the number of photons emitted per solid angle and per surface unit (the X-ray beam brilliance). These two factors contribute equally to the increase of the coherent flux of the X-rays produced because, for an intrinsically incoherent source as a synchrotron accelerator, the coherence of the X-ray beam is directly proportional to the source size and inversely proportional to the distance at which it is measured^5^. The expected increase of coherent X-ray flux, depending also on X-ray energy and source power, can be up to a factor of 200 in the 6–10 keV energy range^5,6^. This increase is expected to open new perspectives for coherent X-ray based experimental approaches such as coherent diffraction imaging. It is certainly of major importance for crystal microscopy, which is—in essence—a highly-coherent photon hungry method^7^. X-ray crystal microscopy, which provides crystalline information of materials, plays a central role in material science and related disciplines (e.g., in physics, mechanics, biology, chemistry, etc.). It combines the high penetration power of X-rays with a 3D description of the crystalline properties at the nanoscale spatial resolution, providing imaging capabilities fully complementary to electron beam diffraction-based microscopy methods.

Coherent diffractive X-ray imaging is a particularly elegant form of X-ray microscopy^8,9^: it circumvents the spatial resolution limit imposed by the comparatively poor focusing power of X-ray optics by direct numerical inversion of the measured intensity diffraction patterns. It can be translated into a form of crystal microscopy by performing measurement of the diffracted signal in the vicinity of a Bragg diffraction peak^10^. The use of a coherent beam results in diffraction patterns composed of fine and high-contrast features, so-called speckles, from which lattice strain and tilt information can be extracted^10^. The most mature of these approaches is the finite-support based Bragg coherent diffraction imaging (BCDI)^10^, which has produced spectacular results for systems also in electrical^11,12^, chemical^13^, and mechanical^14^ environments. BCDI benefits from a small set of experimental constraints: the illumination has to be either perfectly known or approximated to a plane wave in the vicinity of the sample, the 3D information is obtained by rotating the sample over a limited angular range (1–2°) around a diffraction peak, and the intensity patterns have to be acquired with a resolution capable of capturing the details of the speckle pattern^8,10^—generally referred to as “oversampling”. Importantly, BCDI is also quite robust to sample position instabilities. There are, however, some drawbacks: the sample has to be of finite size, typically in the range 0.1–1 μm, in order to fulfill the conditions imposed by the limited X-ray beam coherence and oversampling requirements. In addition, weak or highly inhomogeneous strain fields are extremely challenging to retrieve^15^. The advent of 4^th^-generation synchrotron sources is expected to relax some of these constraints by providing more coherent flux, at least to a certain extent. However, finite-support BCDI studies will remain limited to isolated nano- or micro-metric crystals. Bragg ptychography has been proposed as a suitable alternative to image a large variety of laterally extended and inhomogeneous crystalline samples^16–23^. The use of a finite illumination probe scanned across the sample, is introduced to decompose the inversion problem into a series of easier problems, provided the sample regions illuminated in adjacent beam positions overlap to some extent on the sample surface^24–26^. This results in microscopy with an improved performance profile, capable of imaging weak or highly inhomogeneous strain fields in extended crystals^19,20^. However, while many synchrotron beamlines are already inviting Bragg ptychography experiments, only a few groups have demonstrated successful experiments^18–23^. The extreme sensitivity to instabilities and the strong demands on the setup (including control of the beam position with nanometric accuracy while performing the angular scan), as well as the associated time-consuming data acquisition, are major limits that have hampered the broader application of this powerful tool, to date.

In this article, we investigate the new possibility offered by 4^th^-generation synchrotron x-ray beams with respect to this highly demanding Bragg ptychography crystal microscopy and compare the new result with its counterpart at a 3^rd^-generation synchrotron source.

## Results

**Fig. 1:**
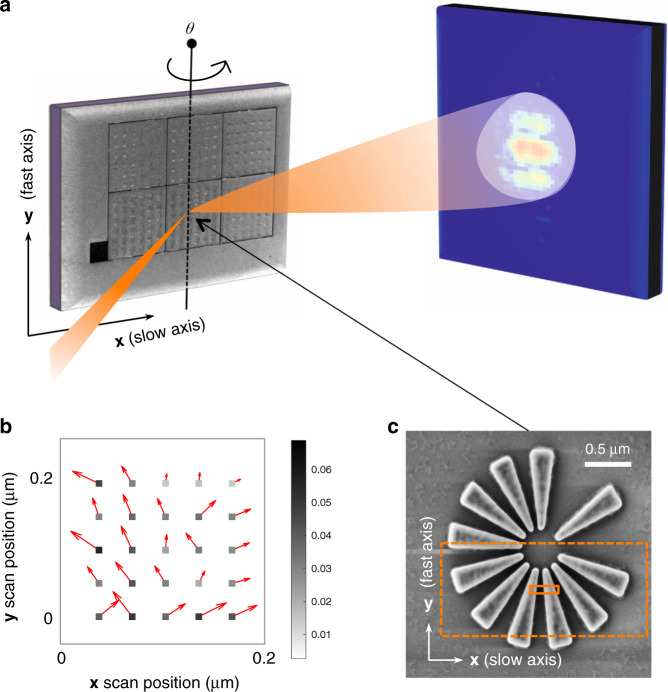
Bragg ptychography experiment. **a** Data acquisition scheme, showing the sample in Bragg condition, illuminated by a focused beam and producing a coherent diffraction pattern, measured by a 2D detector. For the Bragg ptychography data acquisition, the sample is scanned along the **x**- and **y**-axis and further angularly scanned along the *θ* rotation. **b** For this experiment, the scanning stage was not optimized and a strong discrepancy was observed between the nominal **x** and **y** position (square) and the achieved positions (indicated by the red arrows and by the color scale, in μm). **c** SEM picture of the investigated Si-star sample (top view). The orange rectangles represent the footprint of the intensity central lobe (FWHM) on the sample surface (continuous line) and the full dimension of the retrieved probe, including its tails (dashed line)

**Fig. 2:**
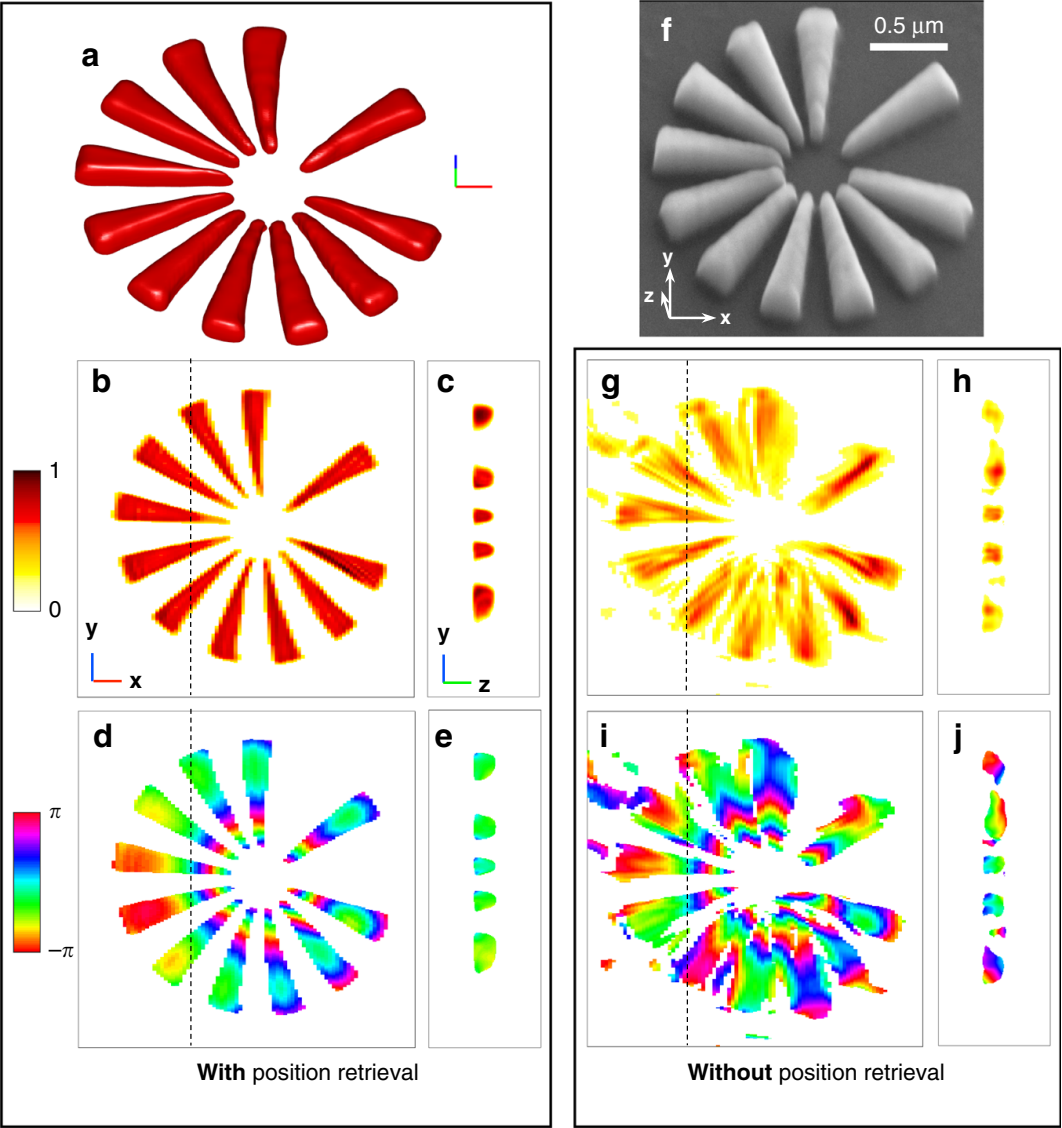
3D Reconstruction of the crystalline Si-star. **a** 3D volume rendering (iso-surface) of the amplitude of the retrieved modulus image. **b**, **c** 2D cross-sections of the 3D amplitude map: **b** is taken at the half-height of the sample structure, while **c** is taken along the dotted line shown in **b**. The amplitude linear color scale is shown in arbitrary units. **d**, **e** 2D cross-sections of the 3D phase map, taken in the same planes as the ones shown in **b** and **c**, respectively. The phase color scale is shown in radians. **f** SEM picture of the same Si-star sample, to be compared to the iso-volume shown in **a**. **g**–**j** Same as **b**–**e**, without introducing the spatial position retrieval. In **a**–**c**, the length of each segment of the 3D frame represents 200 nm

The Bragg ptychography experiment presented here was performed at the NanoMAX beamline of the Max IV Laboratory, Sweden, using a Si-star test chart specifically designed to evaluate the performances of the beamline (see Fig. 1, Materials and methods and Fig. S1 in Supplementary Information (SI)). A 12 keV monochromatic beam was focused to about 80 nm (full width at half maximum of the central lobe intensity) onto the sample plane, producing an illumination spot with ~6 × 10^9^ photons·s^−1^ at the sample position. All technical details and experimental parameters are provided in Materials and methods, as there is no novelty in the data acquisition approach^27^. The sample is a lithographically-patterned crystalline Si-star (Fig. 1), with a nominal thickness of 180 nm and the Si-(110) direction perpendicular to the sample surface. The 2 μm diameter star is composed of 11 branches each 760 nm long, most of them equi-angularly spaced. Close to its center, the branch width and the branch-to-branch distance taper down to 55 nm. The 220 Bragg reflection was chosen for the experiment. In order to fully benefit from the performance of the source (high flux), the ptychography data set was acquired by scanning the sample line by line in a continuous scanning (or fly-scan) mode along the direction perpendicular to the scattering plane (**y**) and in step mode for the direction contained in the scattering plane (**x**) at several angular values *θ*. The exposure time for each frame was fixed to 33 ms, resulting in a total exposure time of about half an hour for the full (2 + 3)D data set, with a total of about 50,000 points. Additional details of the Bragg ptychography acquisition parameters are given in the Materials and methods section, including the prior characterization of the beam using forward ptychography (shown as Fig. S2, in SI). The onset of radiation damage was carefully tracked by observing the change of the diffraction patterns during continuous exposure at several fixed positions on the sample. The Bragg ptychography acquisition time was then limited to about 50% of this threshold (as further illustrated in Fig. S3, SI). No specific effort was made to avoid spatial drift of the sample during the data acquisition which, at the time of the experiment, was performed with a far from optimized setup. Consequently, global drifts and scanning distortions were expected and clearly shown in the data set (see preliminary analysis of the data set in Fig. S4, SI). In this situation, the application of the classical Bragg ptychography inversion process, as described elsewhere^28^, led to a strongly degraded sample image (Fig. 2 and Fig. S5 in SI). The reconstruction is only slightly improved upon the application of a global drift correction between datasets obtained at two successive angular steps, using a cross-correlation comparison (Fig. S6 in SI). The comparison to the reconstruction obtained with the new inversion scheme proposed here shows that much more information can be retrieved from this data set (Fig. 2 and Fig. S7 in SI), as described below.

**Fig. 3:**
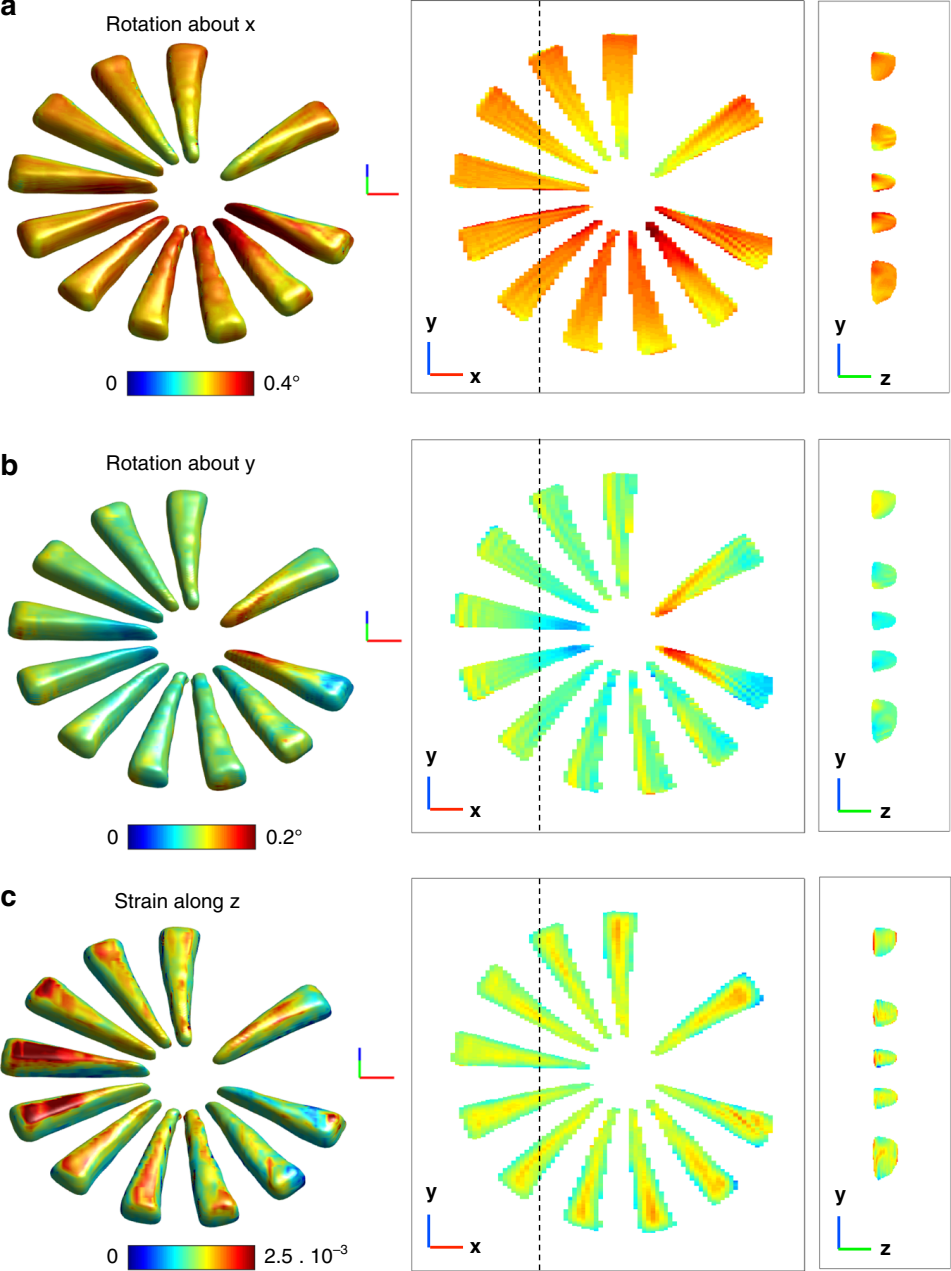
3D crystalline properties extracted from the retrieved phase map. **a**, **b** Rotation of the 220 lattice planes, about the **x** and **y** axes, respectively. The rotation color map angle is shown in degree. **c** Strain of the 220 lattice planes. The strain color map is shown in arbitrary units. In **a**–**c**, the left column corresponds to a 3D rendering of the map onto the volume defined in Fig. 2a while the middle and right columns are defined as in Fig. 2b, c, respectively. The length of each segment of the 3D frame represents 200 nm

Inspired by previous works which proposed to overcome the image degradation due to sample positioning issues in ptychography^29^, we have developed a 3D Bragg ptychography inversion scheme that includes sample position refinement and accounts for fly-scan data acquisition mode. The full strategy is described in detail in the Methods section and illustrated in Figs. S8 to S11 in SI. The quality of the sample image plotted in Fig. 2 is striking. These amplitude and phase maps of the retrieved 3D complex-valued image link, respectively, to the electron density of the crystalline sample and the projection of the crystalline displacement field along the [110] direction^10^. The overall shape (density contour) of the star pattern is retrieved with high definition, which reveals small defects in the patterning that are confirmed by the SEM image of the sample surface in Fig. 2f. The inhomogeneity of the phase map also reveals strain and tilts of the lattice planes, which likely result from the patterning process. Indeed, the phase variation is mostly radial and follows the different branches composing the star. Accessing the phase and, thereby, the displacement field in 3D allows extraction of the strain and lattice tilts of the (110) crystal lattice planes, following the expressions given in reference (27) and shown in Fig. 3. In absence of an absolute reference value for the strain and lattice tilts, we provide in Fig. 3 the relative strain and relative lattice tilts (in degrees). Note that a positive strain value means that the crystal is locally stretched with respect to regions of smaller strain values. The tilt maps exhibit symmetric rotations about the **x**-axis for the branches aligned along the **y**-axis and likewise a rotation about the **y**-axis, for the branches aligned along the **x**-axis. This corresponds to a small distortion (of about 0.1°) of the smallest part of the branches in the vicinity of the star center, which are all slightly curved away from the wafer. The strain map shows a nominally homogeneous strain distribution inside the individual branches. However, the branch surfaces perpendicular to the wafer present a visible strain gradient of about −0.7 × 10^−3^ with respect to the branch core, while the top surface shows a positive strain up to a value of about +0.5 × 10^−3^, as seen on the left branches of Fig. 3c. Interestingly, a strained layer (of about +1 × 10^−3^ with respect to the branch core) is also observed at the Si pattern/wafer interface, in full agreement with previous observations on the same kind of structure using Bragg ptychography and transmission electron microscopy^30^. For this data set, the 3D spatial resolution was estimated using a Fourier shell correlation (FSC) analysis, performed along the three axes of the frame. The two 3D datasets were generated by splitting the angular series into two halves and the axis-sensitive FSC analysis was performed by employing a cylindrical mask along each axis^20^. We found an anisotropic resolution of about 23, 7, and 12 nm along the **x**, **y**, and **z** directions, respectively (see Fig. S12 in SI). Note that the retrieved thickness of the pattern (about 160 nm) is much smaller than the width of the support used during the inversion. This ensures that the support is not impacting the estimation of the spatial resolution along **z**. Together with the sample scattering function, the probe is retrieved using multi-mode decomposition (Fig. S2 in SI) and compared to the one obtained from the preliminary probe analysis, carried out by the standard forward ptychography method. For the first mode, the agreement between the profiles retrieved from forward and Bragg ptychography is clear and further evidenced by the 1D cross-sections plotted in Fig. S2c, d, respectively (SI). The second and third modes retrieved from the Bragg ptychography data set agree fairly well with the ones retrieved by the forward approach. However, a difference is observed in the mode power distribution itself. For the Bragg data set, a larger contribution of the first mode (about 90% for the Bragg data set against 80% for the forward approach, Fig. S2e in SI) and a smaller contribution of the four next ones, are clearly observed. Our hypothesis is that this is caused by the different photon statistics in the two datasets (the Bragg diffraction interaction produces a limited number of photons with respect to the forward scattering approach). To validate this hypothesis, we have performed a numerical study (Fig. S13 in SI). Using the reconstructed probe modes and the object from the forward experiment, we simulated six datasets with different photon count statistics (from noise-free to maximum photon counts down to 3 × 10^1^ photons) and performed a reconstruction using the multi-mode decomposition. The noise-free pattern exhibits blurred interference fringes, which result from the partial coherence effects. The power distribution of the reconstructed probe modes from these datasets does indeed change with the photon statistics. The general trend shows that the lower the photon counts, the more power is allocated to the first probe mode, which indicates less partial coherence effect or a higher degree of coherence. This is not surprising if we look at the diffraction patterns from these different datasets. The decrease of the signal-to-noise ratio results in the observation of more and more zero intensity values, in particular in regions of low-intensity levels, such as the minima between interference fringes. It leads to an artificial increase of the contrast for fringes, which would otherwise be blurred due to partial coherence. Therefore, when the noise level is high the effect of partial coherence becomes less visible in the measured data. It is interesting to note that in this numerical test, when the photon statistics match the Bragg data set signal statistics (i.e. maximum photon counts is 3 × 10^2^ photons), the probe modal power distribution matches well with the one reconstructed for the Bragg data set, as shown in Fig. S2e in SI. Finally, the positional mismatches between the nominal and the optimized sample positions are retrieved and shown in Fig. S14 in SI. The mean value of the global drifts was estimated to be 127 nm, while the local drift mean values typically lie in the 30–40 nm range.

## Discussion

Despite its development in the last 10 years, wide application of Bragg ptychography microscopy has long been inhibited by the demanding experimental requirements, which need to be met to gather a suitable x-ray data set from which a high-fidelity crystalline image can be retrieved; namely position stability at different angular positions. The difficulty of this task is specifically linked to the Bragg case: the Bragg diffraction geometry often requires the ptychography translation stages to work outside an equilibrated position (e.g., involving an inclined stage) and the low Bragg peak intensity makes it difficult to compensate some potential experimental errors, for example, the drifts caused by a fast scanning strategy (short acquisition time). Here, we have demonstrated that the increased flux achievable at 4^th^-generation sources allows the retrieval of high-quality 3D information even under sub-optimal experimental conditions, including significant positioning errors and the use of fly-scan mode. We note that some faint distortions are visible on the shape of the retrieved star (Fig. 2a, b), whose origin is not clear. On the one hand, none of the crystalline characterization approaches we are aware of is able to provide a 3D description of this Si pattern with the required sensitivity and resolution to confirm the validity of these features (EBSD characterization was performed, but did not provide convincing results). On the other hand, none of the simulations we performed produced similar artifacts (even when a strained star pattern is considered, not shown). In this context, we cannot fully exclude the possibility that these crystalline distortions are real.

**Fig. 4:**
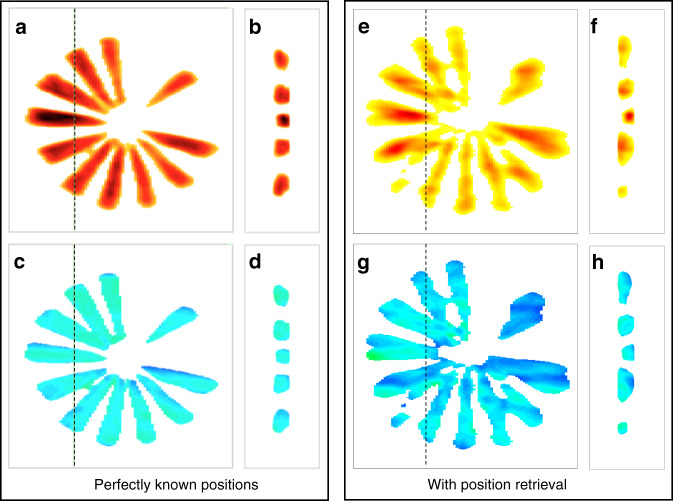
Impact of the intensity level on the inversion quality (numerical study). The 3D Bragg ptychography data set was simulated with 100 times less intensity (maximum of three photons per pixel) with respect to the experimental data and poised with Poisson noise. The sample is strain-free (constant phase) and the scanning parameters are the same as the ones of the experimental data set. In **a**–**d** no spatial drift was considered (perfect setup) and a step-scan mode was introduced to generate the data. In **e**–**h,** spatial drifts and fly-scan were introduced to generate the data and the new inversion approach was used to retrieve the object. The comparison with **a**–**d** shows a strong degradation of the image, due to the impossibility to retrieve in a satisfactory manner the object, the probe, and the sample position, as a result of a lack of information in the data set (limited intensity level)

**Fig. 5:**
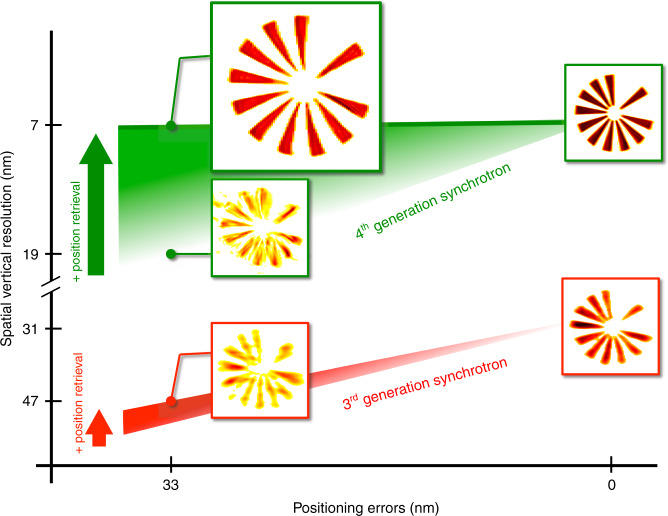
Summary of the Bragg ptychography performances. The graph synthesizes the gain on the image quality (spatial resolution along the vertical direction, in nm) obtained in this study as a function of the performances of the setup (positioning errors, in nm) when one introduces a position retrieval routine in the inversion scheme. With a 3^rd^-generation synchrotron source, the gain is limited and the experimental approach only performs well with a reliable sample scanner. On the contrary, with the coherent flux available at the 4^th^-generation synchrotron source, a high (almost maximal) quality image can be retrieved even for non-perfect scanning performances. The illustrations correspond to the density distributions presented in the main text and in Supplementary information

In order to further clarify the advantage of using 4^th^-generation synchrotron sources for Bragg ptychography, we have performed a series of simulation tests. The reference image, shown in Fig. S15 in SI, was obtained using reconstruction from a perfect data set (including the fly-scan mode), for which the intensity range has been set to match the dynamic range observed in the experiment (i.e., a maximum of 300 photons per pixel). In this reference reconstruction, neither noise nor distortion is expected. It can be compared to the reconstructions presented in Figs. S16 and S17, which introduce Poisson noise and step-scan mode, respectively (SI). Note that the reconstructions obtained from these two last tests show only marginal differences with respect to the reference. Remarkably, when the data set is strongly degraded by random spatial drifts (as illustrated in Fig. S5 in SI), it is still possible to extract a high-fidelity image of the sample, as evidenced by the reconstruction shown in Fig. S8 in SI. For comparison, a data set 100 times less intense, mimicking the performances of a third-generation source was generated and fed into the inversion algorithm assuming a perfect acquisition scheme, i.e., with no drifts. The retrieved image (Fig. 4a–d) is visibly degraded with respect to the one shown in Fig. S8 (i.e., resulting from the degraded data set acquired with the high dynamical range). The degradation is even worse when spatial drifts are also introduced into the low-intensity data set as shown in Fig. 4e–h. This simulation demonstrates the difficulty in extracting a high-quality image from a low-intensity data set, where there is an almost complete lack of the high-frequency information, that would ideally be present in the low-intensity region of the diffraction patterns. On the contrary, when high-intensity data are accessible, even if obtained under sub-optimal scanning conditions, the information contained in the high-frequency region of the diffraction pattern is robust enough to allow for the object, the probe, and the true spatial scan positions to be retrieved, as summarized in Fig. 5. This is an important result, which shows that the position refinement method, introduced here for the first time in the BP framework, is unlikely to offer significant improvements at 3^rd^-generation synchrotron sources but can provide a direct improvement of the image quality (accuracy and spatial resolution) here. The limit between these two regimes can be assessed from the experimental data set, by reducing the amount of data used in the analysis: the quality of the image is still preserved if we use only half of the data set, i.e., a two times larger angular step. It starts degrading only when two-thirds of the data set is removed (see Figs. S18 and S19 in SI). This shows that a high-quality image could still be obtained if half of the diffracted photons are available (i.e., for a total acquisition time of about 15 min), but not below this limit. Consequently, if the same unoptimized setup is used at a third-generation source, the much lower available coherent flux would require an increase in acquisition time by a factor of about 100, leading to a measurement lasting for about 25 h, a strategy that would be totally irrelevant to scientific applications.

Finally, we would like to highlight the gain made possible with this approach at a 4^th^-generation synchrotron source. Although a direct comparison with other experiments (for which the investigated sample and the experimental setup are different) is challenging, it is, however, interesting to compare the present result with previous results at 3^rd^-generation synchrotron sources. To this end, we choose a result obtained by some of us at ID13-ESRF^30^, on a rather comparable Si pattern. Table S1 (SI) summarizes the main characteristics of the two experiments and the respective retrieved image properties. These include the coherent flux at sample position *F*, the retrieved field of view *FOV*, the 3D spatial resolution, and the acquisition time *t*. While for the coherent flux *F*, the gain is about 60, it is interesting to note that a 16 times larger *FOV*, obtained with 17 times improved spatial resolution is achieved for 10 times shorter acquisition time. This comparison further highlights the gains made possible by the increase in coherent flux and by the inversion strategy (i.e., probe retrieval and position retrieval).

This first Bragg ptychography experiment at a 4^th^-generation synchrotron source demonstrates not only the possibility to speed up data acquisition by shortening the acquisition time, as expected from the source’s high brilliance but also the possibility to work in a new high dynamic range regime in which experimental constraints are strongly relaxed. We are confident that the advent of 4^th^-generation synchrotron sources will make this crystal microscopy compatible with a wider range of experimental set-ups and applicable to a wider range of scientific questions. Finally, the huge improvement in acquisition time paves the way for future time-resolved studies.

## Materials and methods

### Sample preparation

The Si-star was patterned onto a Silicon-on-insulator (SOI) wafer, composed of three layers: a crystalline top Si (110) layer (with a nominal thickness of 0.18 μm), an intermediate amorphous SiO_2_ layer (0.02 μm), and a Si (001) substrate. The star design was defined following two main constraints: the design should contain features presenting a large range of sizes to test the spatial resolution performances and it should contain a large volume of scattering elements (i.e., Si crystal) to optimize the diffraction signal. The star patterning was performed at Myfab Chalmers (Sweden). In order to fabricate the locally dense, high aspect ratio and high-resolution structures, the SOI wafers were coated with a 40 nm thick Hydrogen Silsesquioxane resist, exposed using electron-beam lithography, and developed in a Tetramethylammonium Hydroxide based developer (0.237 N). The resist structures were annealed at 900 °C in an N_2_ atmosphere using rapid thermal processing to increase their durability in the plasma etching process. The pattern transfer was achieved by a highly directional chlorine plasma etching process using an inductively coupled plasma etcher, yielding an etch selectivity of nearly 5 and perfectly vertical sidewalls. Ultimately, features as small as 20 nm and separated by 15 nm could be obtained. The crystallographic orientation mismatch between the top Si layer and the Si substrate enabled to image solely the Si top structure with x-ray Bragg diffraction (Fig. 1 and Fig. S1 in SI).

### Experimental approaches

#### Scanning electron microscopy characterization

The scanning electron microscopy analysis (Fig. S1, SI) was performed with a Zeiss Gemini500 ultra-high resolution field emission electron microscope. For secondary electron (SE) imaging, this system is equipped with an in-lens detector, ideal for displaying surface structures (it detects SE electrons directly in the beam path), and a SE lateral detector, which emphasizes the topography of the specimen. SE lateral images (Fig. S1b) were obtained at 15 kV with a 45°-sample tilt and a 14.7 mm working distance. In-lens images (Fig. S1c) were acquired at 1 kV and a very short working distance (1.4 mm) in order to improve the signal-to-noise ratio and to avoid charging effects. The resolution at an optimum working distance is 0.6 nm at 15 kV and 1.1 nm at 1 kV. The incident probe current was measured using a Faraday cup (1.19 nA for 15 kV and 49.2 pA for 1 kV).

#### X-ray experimental setup

The x-ray beam of the NanoMAX beamline was filtered with a horizontal double-crystal monochromator (Si 111, bandwidth of ~10^−4^) to provide a monochromatic beam with energy of 12 keV (or wavelength λ of 1.0325 Å). In order to select a spatially coherent beam for the experiment, the secondary source aperture (SSA) slits placed at 47 m from the sample stage^5^ were set to a width of 250 μm, in both horizontal (H) and vertical (V) directions. A set of fixed-curvature Kirkpatrick–Baez mirrors was used to focus the beam onto the sample. It provides a numerical aperture of 6.4 × 10^−4^, in both directions. From a dedicated beam characterization (using forward ptychography, see below), the beam profile was extracted and the central spot size (FWHM of intensity) was estimated to be about 80 nm (resp. 90 nm) along the vertical (resp. horizontal) direction. For the Bragg ptychography data acquisition, a 2D Merlin detector (515 × 515 pixels with a pitch size of 55 µm^2^ × 55 µm^2^) was mounted on a robot arm. The sample-to-detector distance was set to 1.2 m, a distance large enough to ensure the oversampling of the diffraction pattern.

#### Data acquisition

The specular 220 Bragg reflection of the patterned Si (110) layer was chosen. It corresponds to a Bragg angle *θ*_*B*_ = 15.6°. The sample was placed horizontally onto a piezo translation stage, allowing for nanometric motion along the **x**, **y**, and **z** directions, as defined in Fig. S1a in SI. At the Bragg angle, the beam footprint is elongated as schematically shown in Fig. S1c (orange rectangle). The translation stage was fixed onto a two-circle diffractometer, used to perform the angular rotation of the sample perpendicularly to the Bragg vector **G** and a vertical scattering geometry was chosen (i.e., the incident and diffracted beams are contained into—or close to—the vertical plane). The 2D pixel detector was placed at an angle of 2*θ*_*B*_ with respect to the direct beam direction. The 3D diffraction intensity distribution was acquired as a function of the **q** wave vector transfer (defined by **q** = **k**_f_ − **k**_i_, with **k**_i,f_ the incident and diffracted wave vectors, respectively). The two first components of **q** correspond to the pixels rows and columns of the 2D detector, while the third direction is investigated by scanning the incidence angle along the *θ* rotation (defined by the rotation axis perpendicular to the scattering plane) in steps of *δθ*. In Bragg conditions, **q** exactly matches **G**. At each angular position, a square grid of 41 × 45 positions has been scanned in continuous (or fly-scan) mode along **y** and stepping mode along **x**. The nominal step size along both directions was set to 50 nm, small enough to ensure enough overlap from one illumination position to the other. Note the spiral scan approach often used in ptychography was not available at the beamline, in the fly-scan mode. However, the uncertainty in the positioning introduces enough fluctuations with respect to a perfectly periodically scan to avoid the artifacts usually observed in forward ptychography^31^. The third direction of the reciprocal space was investigated by scanning the sample angularly along the so-called rocking curve (*θ* direction). Along this direction, a scan of 27 angular positions, covering a range of 0.42° (i.e., angular step *δθ* of 0.016°) was designed. At each spatial and angular sample position, a 2D diffraction pattern was acquired, with an exposure time of 33 ms per frame. Two diffraction patterns are displayed in Fig. S1d, e, evidencing the large available coherent flux.

#### Beam characterization

Before the Bragg ptychography experiment, the beam profile was characterized using forward transmission ptychography. A gold test pattern was used and positioned upstream the focal plane at a distance of 500 µm. A step scan over a square grid of 16 × 16 positions with a step size of 133 nm was carried out. A Pilatus 100 K detector (487 × 195 pixels with a pixel size of 172 µm^2^ × 172 µm^2^) was placed downstream at a distance of 4.01 m from the sample. Each frame was exposed for 100 ms.

A sub-region of 170 × 170 pixels of the diffraction patterns, centered on the forward beam, was used for the reconstruction. The used reconstruction algorithm was the standard ePIE^32^ with five probe modes^33^. The initial guesses for the probe modes were generated by multiplying a simulated beam profile based on the experimental configuration model with five different random matrices. The initial guess for the object was simply a matrix of unities (1 s) with a flat phase. The reconstruction was run for 200 iterations. The reconstructed probe modes were then numerically propagated to the focal plane. The first three probe modes containing 94% of the total power are shown in Fig. S2a in SI. Only amplitude parts of modes #2 and #3 are shown in a gray color map to highlight their mode structures. It should be noted that this beam information (probe profiles and mode distribution) was not used for the Bragg ptychography reconstructions.

### Bragg ptychography inversion details

The main specificities of the new inversion scheme are outlined hereafter. Regarding the retrieval of the sample positioning, the major difficulty comes from the fact that the 3D illuminated sample volume (defined by the beam profile and the beam-to-sample position) is related to the 3D intensity pattern by a 3D Fourier transform relation. It implies that the whole set of 3D Fourier components is associated with a single beam-to-sample position. This holds true for the two first dimensions, which lie in the detector plane, but it becomes invalidated as soon as the sample moves during the angular exploration along the rocking curve. In order to decouple the 3D intensity distribution from the 3D illuminated volume, we took advantage of a recently proposed 3D Bragg ptychography formalism, which makes use of the Radon transform and its inverse counterpart^20,23^. It allows one to link the individual 2D diffraction pattern to a specific tomographic 2D projection of the 3D sample at a given position. Thereby, each individual 2D diffraction pattern can be associated with a single specific sample position. Once this is introduced, the refinement of the sample position can be performed on the full data set. As it is based on correlations between diffraction patterns, this approach still works for samples that would exhibit crystalline distortions only (i.e., no electron density contrast). We note that a few different strategies have been proposed in forward ptychography^34^ that should be able to achieve this, but comparing their respective merits in the BP framework falls out of the scope of this study. As extensively explored in forward ptychography^35–37^, the fly-scan acquisition mode can speed up the data collection by saving the overhead time on the de-acceleration and settlement of the scanning motors at each scanning position. However, it blurs the diffraction patterns, which is equivalent to introducing partial coherence effects. Mixed-state reconstruction strategy^33^ needs to be introduced to account for using mode decomposition. Note that additional sources of de-coherence, such as any other vibrations or partial coherence of the beam, are also accounted for in this scheme. This approach allows retrieving a series of effective incoherent probes, the more prominent mode being associated with the beam profile (Fig. S2 in SI). In addition, a regularization term is introduced along the inversion, in order to promote the solutions for which the object thickness is smaller than a certain value^19^. Finally, to further help the reconstruction, a threshold process is introduced. The up-sampling strategy and the probe reconstruction have been first successfully tested on mock datasets for both fly-scan and step-scan acquisition modes (see Figs. S8 and S9 in SI, respectively). Prior to the inversion, the sample thickness is directly estimated from the data set, using an L-curve optimization approach^19^. The same approach is used to refine the film orientation, which exhibits a small tilt with respect to the 110 Bragg vector. The summary of the L-curve analysis is shown in Fig. S10, first tested on simulated numerical datasets (full intensity signal and Poisson noise corrupted data) before it is used on the experimental data (SI). The successful application of this whole inversion scheme on the experimental data set is illustrated in the 3D sample reconstruction shown in Fig. S7, while the limited impact of the threshold constraint on the same data set is confirmed with the reconstruction shown in Fig. S11, SI. The full inversion details are described hereafter.

The inversion strategy is composed of three successive steps:

- data shaping,
- preliminary corrections of global spatial drifts,
- inversion process,

which are described below in details.

- *Data shaping:* For the Bragg ptychographic reconstruction, a sub-region of 400 × 360 pixels, centered on the Bragg peak, is cropped out from the full-size measurement data set. The gap between different sensor modules of the Merlin detector is masked out. The diffraction patterns are then binned by 2 along each dimension (i.e., 2 × 2 pixels are binned into 1 larger virtual pixel of 110 × 110 µm^2^), which gives a dimension of 200 × 180 pixels for each frame of the diffraction patterns. This step is introduced to save data processing time, given the sampling condition is still fully satisfied after the binning operation. This 3D measurement volume, in the reciprocal space, determines the pixel sizes in its conjugate 3D real-space; they correspond to 26.6, 6.3, and 5.3 nm respectively along **x**, **y**, **z**.
- *Preliminary corrections of global spatial drifts:* In order to partly correct for spatial drifts and misalignments, we introduce a numerical approach, able to mitigate the misalignment between the data acquired at different angles. It corresponds to a spatial re-alignment of the sample for each angle, performed *via* building spatial intensity maps (SIM). The SIM is a 2D map produced for each rocking angle by assigning to each spatial scanning position the sum of the intensities integrated into the detector plane. As the center of the rocking curve (angle #14) has the highest intensity signal, the cross-correlation between the SIM in a given angle and the SIM for angle #14 is used to perform a spatial registration along the rocking angles. From these aligned SIMs, a sub-region (of 41 × 45 ptychographic spatial positions) is set (still covering the whole star) and the corresponding diffraction patterns from these selected scanning points were used for reconstruction.
- *The inversion process:* The inversion part contains several numerical routines used cyclically and/or simultaneously. They aim for:
  - retrieval of probe and object
  - angular up-sampling,
  - regularization of the sample thickness,
  - incoherent background fitting,
  - global and individual drift corrections.

These routines are individually detailed hereafter before the full inversion process used for the inversion of the data set is described.

- Retrieval of probe and object: A recently developed reconstruction strategy^28^ is used that allows for the simultaneous retrieval of both object and probe; such a probe retrieval is enabled in Bragg geometry by enforcing an invariance constraint along the propagation direction of the beam. This constraint is well satisfied here, since the depth of focus of the focusing optics (about 120 μm at this energy) is much larger than the intersected volume with the object, due to their small numerical aperture. To account for the loss of coherence in the measured signal along the fly-scan direction, five probe modes are used^33^. For the object, only one mode is used, and the chosen initial guess is a uniform slab with a thickness of 190 nm.

The sampling along the angular rocking direction is up-sampled by a factor of two^28^ and the reconstruction is performed in the orthogonal frame defined by the detector plane and the exit beam direction **k**_f_; this strategy allows us to enforce the reciprocal-space constraints (i.e., the agreement between the measured and the estimated intensity patterns) without any interpolation of the measurements inherently acquired in a non-orthogonal frame (see Fig. S1). This direct mapping of the real and reciprocal space frames is enabled via a modified 3D Fourier transform recently developed^38^, which still preserves the counting statistics of the measured signal.

- Angular up-sampling: The angular step size determines the real-space window size along **x** and **z** due to the inclined geometry and according to the Nyquist sampling theorem. To ensure the whole illuminated volume is included in the window size (i.e., satisfying Nyquist sampling condition), a smaller angular step size as the one used for the data collection in the experiment is needed. To reduce the step size, we artificially insert virtual angular points between the measured ones, i.e., computationally up-sampling the angular dimension. During the inversion, those virtual angular information can be retrieved^28^.

- Sample thickness regularization: Thickness-support regularization is applied to avoid the inherent under-determination along the beam propagation direction due to lack of diversity^19^. To find the optimal thickness for the support constraint, we employ the L-curve method^19,39^. In this way, a small tilt (of about 1.6°) around the **x***-*axis is found between the star (**x, y**) plane and the translational scan plane. To determine the tilt value, we run a series of reconstructions with different tilt values and select the value with the smallest reconstruction error. The methods to determine the thickness and the tilt were carefully studied and previously validated via numerical simulations (Fig. S10, SI). The thickness, fixed to 190 nm, is slightly larger than the nominal thickness of the Si layer.

- Incoherent background fitting: an incoherent background contribution is jointly fitted during the inversion to account for incoherent photon counts^28^, supposedly induced by air scattering.

- Global and individual drift corrections: The preliminary corrections of the global drift between the angles described above do not account for additional drifts within each 2D map acquired at a given angle. It results, indeed, in limited reconstruction quality. To further improve the reconstructions, drift correction during the inversion needs to be introduced. To this end, each diffraction pattern needs to be registered to a specific spatial position, which is only possible thanks to the back-projection reconstruction strategy, which decouples the 2D individual diffraction patterns from the 3D intensity distribution at a given spatial position^38^. The drift correction includes two parts: the global drift correction between angles and the correction of the individual drifts within each angle. The global drift correction (i.e., for each angle) is derived from the cross-correlation between the SIMs derived from the current reconstruction and from the measurements. Within each angle, the individual drift correction is performed with the annealing method^29^. It should be noted that cross-correlation-based registrations provide sub-pixel drift estimates with an accuracy only restricted by the signal-to-noise ratio^40^.

For the inversion, 200 iterations were first run using the ptychography iterative engine (i.e., ordered subset with Gaussian noise model). It included the retrieval of probe and object, the sample thickness regularization, and the incoherent background fitting. To account for spatial drifts, the iterative reconstruction relying on back-projection was run for 400 more iterations: the global drift correction started after ten iterations and was stopped after 200 iterations, immediately followed by the individual drift correction iterations, stopped after 150 iterations (the last 50 iterations were run without any drift corrections). Finally, an amplitude threshold was also implemented for the object reconstruction: during the first 100 iterations, the relative object amplitudes lower than 3% of the maximum were set to zero. Then, this threshold was increased to 10% for the next 200 iterations and for the remaining 200 iterations, the threshold was set back to 3%. As the iterations progress, this strategy allows small-amplitude artifacts to vanish much faster. The iterative background correction and sample thickness regularization were performed during this back-projection-based reconstruction strategy as well.

## Supplementary information

Supplementary Information

## Data Availability

All raw data used in this article are available at 10.5281/zenodo.5892030.

## Code availability

All Matlab routines developed for the Bragg ptychography analysis and used in this article have been deposited in the Zenodo database under accession code 10.5281/zenodo.5892030.

## References

[1] Eriksson M, van der Veen JF, Quitmann C (2014). Diffraction-limited storage rings - a window to the science of tomorrow. J. Synchrotron Radiat..

[2] Tavares PF (2018). Commissioning and first-year operational results of the MAX IV 3 GeV ring. J. Synchrotron Radiat..

[3] Liu L, Neuenschwander RT, Rodrigues ARD (2019). Synchrotron radiation sources in Brazil.. Philos. Trans. A Math. Phys. Eng. Sci..

[4] Raimondi P (2016). ESRF-EBS: the extremely brilliant source project. Synchrotron Radiat. News.

[5] Björling A (2020). Ptychographic characterization of a coherent nanofocused X-ray beam. Opt. Express.

[6] Kahnt M (2021). Measurement of the coherent beam properties at the CoSAXS beamline. J. Synchrotron Radiat..

[7] Stangl, J. et al. *Nanobeam X-Ray Scattering: Probing Matter at the Nanoscale* (Wiley-VCH, 2013).

[8] Miao JW (2015). Beyond crystallography: diffractive imaging using coherent x-ray light sources. Science.

[9] Chapman HN, Nugent KA (2010). Coherent lensless X-ray imaging. Nat. Photonics.

[10] Pfeifer MA (2006). Three-dimensional mapping of a deformation field inside a nanocrystal. Nature.

[11] Ulvestad A (2015). Topological defect dynamics in operando battery nanoparticles. Science.

[12] Singer A (2018). Nucleation of dislocations and their dynamics in layered oxide cathode materials during battery charging. Nat. Energy.

[13] Clark JN (2015). Three-dimensional imaging of dislocation propagation during crystal growth and dissolution. Nat. Mater..

[14] Dupraz M (2017). 3D imaging of a dislocation loop at the onset of plasticity in an indented nanocrystal. Nano Lett..

[15] Minkevich AA (2007). Inversion of the diffraction pattern from an inhomogeneously strained crystal using an iterative algorithm. Phys. Rev. B.

[16] Godard P (2011). Three-dimensional high-resolution quantitative microscopy of extended crystals. Nat. Commun..

[17] Hruszkewycz SO (2013). Imaging local polarization in ferroelectric thin films by coherent X-ray Bragg projection ptychography. Phys. Rev. Lett..

[18] Takahashi Y (2013). Bragg X-ray ptychography of a silicon crystal: visualization of the dislocation strain field and the production of a vortex beam. Phys. Rev. B.

[19] Mastropietro F (2017). Revealing crystalline domains in a mollusc shell single-crystalline prism. Nat. Mater..

[20] Hruszkewycz SO (2017). High-resolution three-dimensional structural microscopy by single-angle Bragg ptychography. Nat. Mater..

[21] Dzhigaev D (2017). X-ray Bragg ptychography on a single InGaN/GaN core–shell nanowire. ACS Nano.

[22] Kim C (2018). Three-dimensional imaging of phase ordering in an Fe-Al alloy by bragg ptychography. Phys. Rev. Lett..

[23] Hill MO (2018). Measuring three-dimensional strain and structural defects in a single InGaAs nanowire using coherent X-ray multiangle Bragg projection ptychography. Nano Lett..

[24] Faulkner HML, Rodenburg JM (2004). Movable aperture lensless transmission microscopy: a novel phase retrieval algorithm. Phys. Rev. Lett..

[25] Thibault P (2008). High-resolution scanning x-ray diffraction microscopy. Science.

[26] Dierolf M (2010). Ptychographic X-ray computed tomography at the nanoscale. Nature.

[27] Pateras AI (2015). Nondestructive three-dimensional imaging of crystal strain and rotations in an extended bonded semiconductor heterostructure. Phys. Rev. B.

[28] Li, P. et al. Revealing nano-scale lattice distortions in implanted material with 3D Bragg ptychography. *Nat. Commun.* **12**, 7059 (2021). 10.1038/s41467-021-27224-5 PMC8642407 34862390

[29] Maiden AM (2012). An annealing algorithm to correct positioning errors in ptychography. Ultramicroscopy.

[30] Chamard V (2015). Strain in a silicon-on-insulator nanostructure revealed by 3D X-ray Bragg ptychography. Sci. Rep..

[31] Thibault P (2009). Probe retrieval in ptychographic coherent diffractive imaging. Ultramicroscopy.

[32] Maiden AM, Rodenburg JM (2009). An improved ptychographical phase retrieval algorithm for diffractive imaging. Ultramicroscopy.

[33] Thibault P, Menzel A (2013). Reconstructing state mixtures from diffraction measurements. Nature.

[34] Odstrčil M, Menzel A, Guizar-Sicairos M (2018). Iterative least-squares solver for generalized maximum-likelihood ptychography. Opt. Express.

[35] Deng JJ (2015). Continuous motion scan ptychography: characterization for increased speed in coherent x-ray imaging. Opt. Express.

[36] Huang XJ (2015). Fly-scan ptychography. Sci. Rep..

[37] Pelz PM (2014). On-the-fly scans for X-ray ptychography. Appl. Phys. Lett..

[38] Li P (2020). General approaches for shear-correcting coordinate transformations in Bragg coherent diffraction imaging. Part II. J. Appl. Crystallogr..

[39] Hansen PC (1992). Analysis of discrete Ill-posed problems by means of the L-curve. SIAM Rev..

[40] Woodward, P. M. *Probability and Information Theory with Applications to Radar* (Pergamon Press, 1953).

