# Supplementary material for: 4th generation synchrotron source boosts crystalline imaging at the nanoscale
Source: Light Sci Appl. 2022 Mar 25;11:73. doi: 10.1038/s41377-022-00758-z (PMC8956681; doi:10.1038/s41377-022-00758-z)
Supplement: Supplementary file 1 — Supplementary Information [file 41377_2022_758_MOESM1_ESM.pdf]

Supplementary Information for

4th generation synchrotron source boosts crystalline imaging at the nanoscale

Peng Li[1†], Marc Allain[1], Tilman Andreas Grünewald[1], Marcus Rommel[2], Andrea Campos[3], Dina Carbone[4], Virginie Chamard[1*]

[1]Aix-Marseille Univ, CNRS, Centrale Marseille, Institut Fresnel, Marseille, France.
[2] Nanofabrication Laboratory, Department of Microtechnology and Nanoscience, MC2, Chalmers University of Technology, SE-412 96 Gothenburg, Sweden.

[3] Aix Marseille Univ, CNRS, Centrale Marseille, FSCM (FR1739), CP2M, 13397 Marseille, France.

[4] MAX IV Laboratory, Fotongatan 2, 225 94 Lund, Sweden.

*Correspondence to: virginie.chamard@fresnel.fr
†Now at Diamond Light Source, Harwell Science and Innovation Campus, Fermi Ave, Didcot OX11 0DE, UK

This document contains Supplementary Figures S1 – S19 and Table S1.

Figure S1. Bragg ptychography experimental set-up and sample description. **a,** Schematics of the sample cross-section and data acquisition scheme. **b – c,** Scanning electron microscopy views (45 ° tilted and from the top) of the Si-star sample. In **c**, the beam footprint is indicated as an orange rectangle for $\theta = \theta_B$. **d – e,** Two diffraction patterns acquired at two different beam-to-sample positions marked as yellow dots and labeled in **c**. The log10 intensity color scale is in photons.

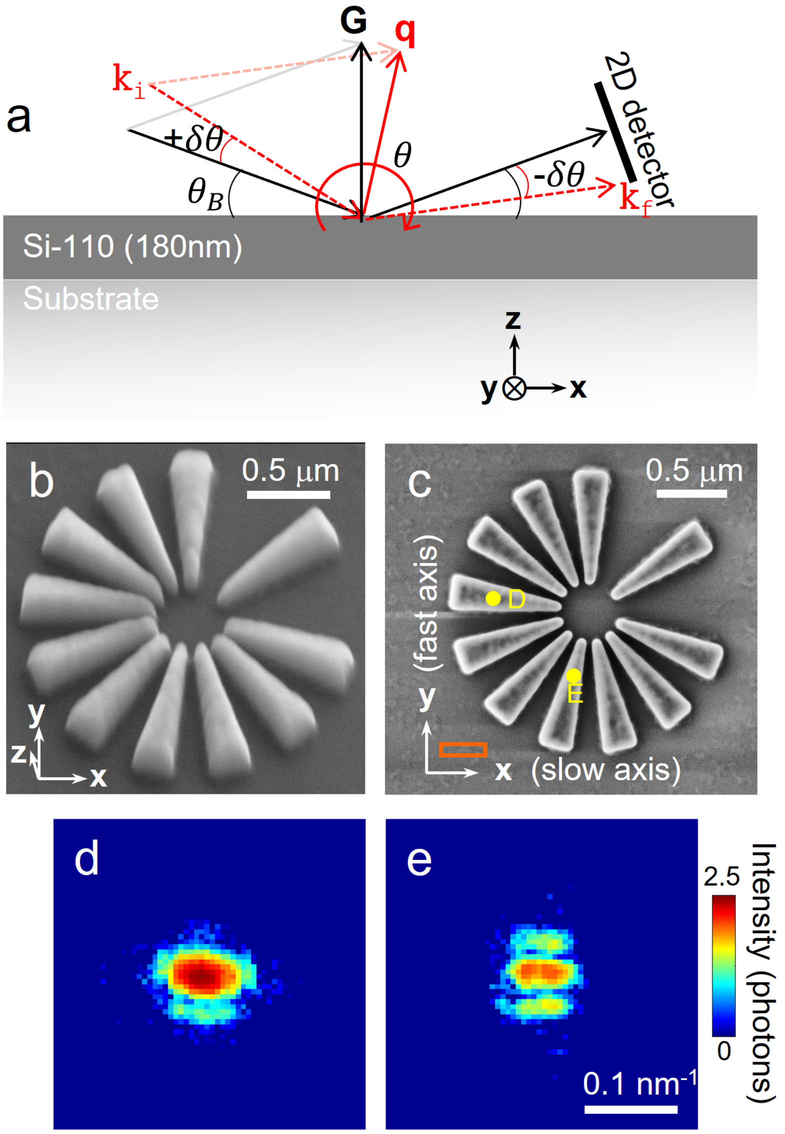

Figure S2. Retrieved x-ray probe using forward ptychography and Bragg ptychography. a, Forward ptychography reconstruction of the probe: the first three probe modes containing 94% of the total power are shown. The phase information is plotted according to the shown color map, in radian, while the brightness corresponds to the amplitude. Only amplitude parts of modes #2 and #3 are shown to highlight their mode structures, following the shown gray color scale. **b,** Same as **a** for the Bragg ptychography data set. **c – d,** Vertical and horizontal 1D cross-sections of the first probe mode shown in **a** and **b**, respectively. **e,** Relative probe mode power distribution for (red) the Bragg ptychography data set, (green) the forward ptychography data set and (blue) the forward ptychography data set where the total number of photons was limited to match to the number photons present in the Bragg ptychography data set. The scale bars represent a length of 200 nm.

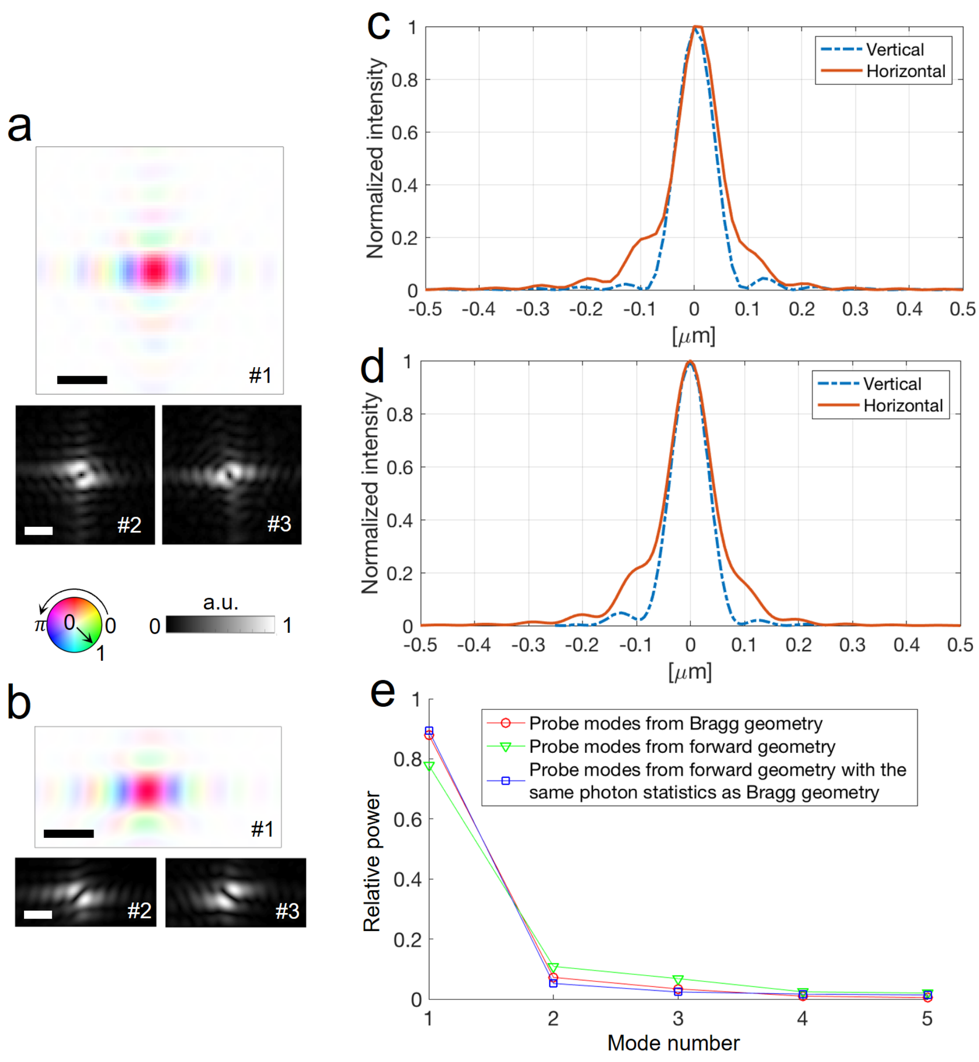

Figure S3. Tracking radiation damage. a, 2D spatial map obtained by integrating the diffracted intensity onto the detector plane (SMP maps), for a given θ value. The Si-star pattern can be clearly identified. **b - c,** Similar to A and acquired after the measurement shown in **a** (resp. **b**), onto the same Si-star sample, at (almost) the same angles along the rocking curve. Despite some differences due to the different acquisition times and the different step sizes, **a** and **b** look very similar, while **c** starts to exhibit some faint differences, likely due to onset of radiation damage. **d,** A series of 2D spatial maps obtained at different θ values, to be compared to the next measurement series shown in **e**. Note that, in **d** and **e**, the acquisition time (indicated on the figure) is not the same. The data shown in **d** are part of the Bragg ptychography data set used for the inversion. The scan number is indicated with a '#' for each 2D data set. These numbers indicate the acquisition order. **f,** Three 2D diffraction patterns extracted from a time series, obtained on a Si nanowire (200 nm large) located nearby the Si star. The exposure time is 1 s, repeated 101 times. No visible modification of the diffraction patterns is observed (the numbers indicate the acquisition order in this time series). **g,** Integrated intensity calculated along the time series shown in **f**.

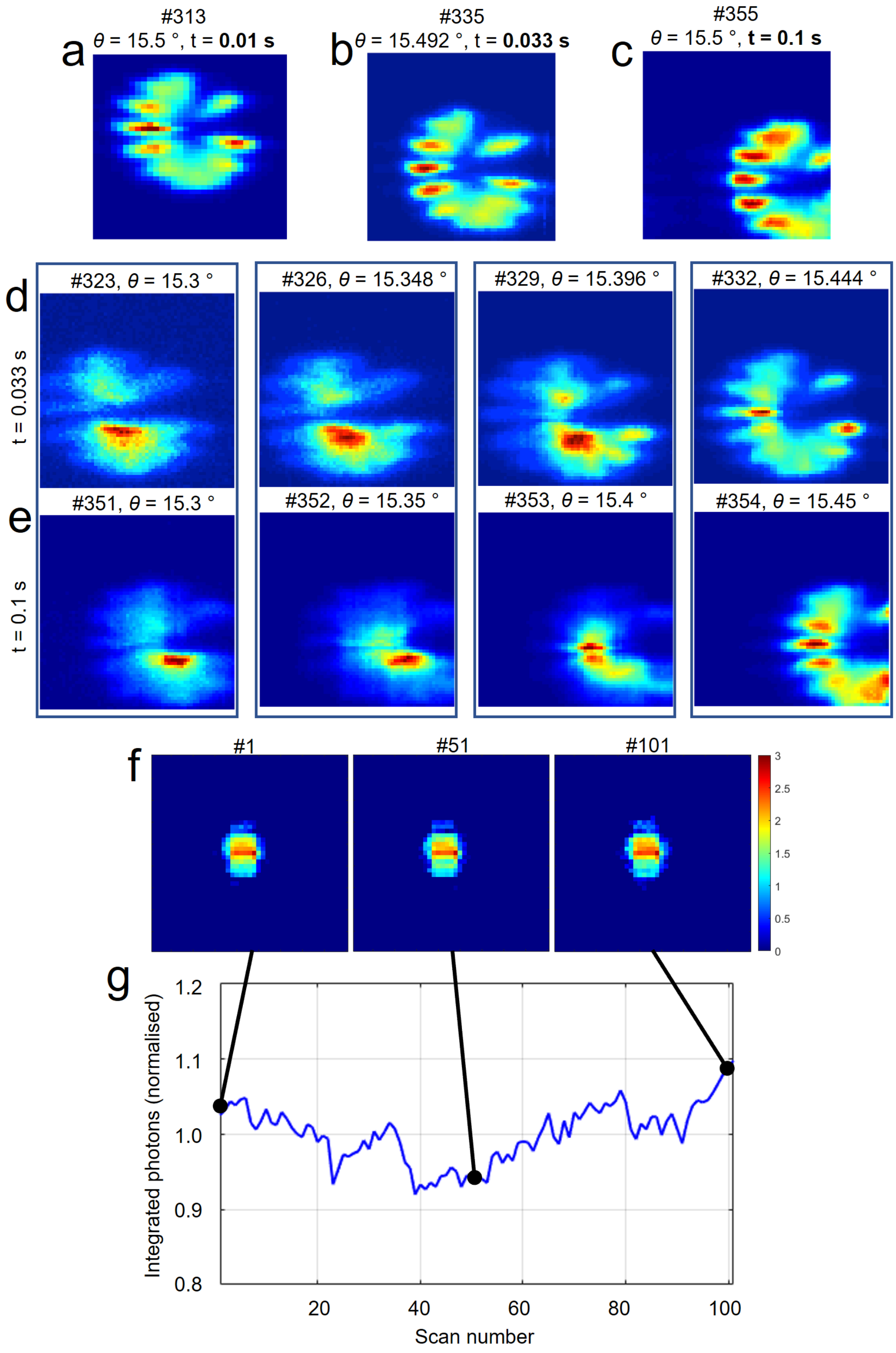

#313
a θ = 15.5 °, t = 0.01 s
#335
b θ = 15.492 °, t = 0.033 s
#355
c θ = 15.5 °, t = 0.1 s
d
t = 0.033 s
#323, θ = 15.3 °
#326, θ = 15.348 °
#329, θ = 15.396 °
#332, θ = 15.444 °
e
t = 0.1 s
#351, θ = 15.3 °
#352, θ = 15.35 °
#353, θ = 15.4 °
#354, θ = 15.45 °
f
#1
#51
#101
3
2.5
2
1.5
1
0.5
0
g
Integrated photons (normalised)
1.2
1.1
1.0
0.9
0.8
20
40
60
80
100
Scan number

Figure S4. Corruption of the data set by spatial drift. Same dataset as in Fig. S3d, with red lines superimposed onto the 2D maps to evidence the global spatial drift observed from one angular position to another.

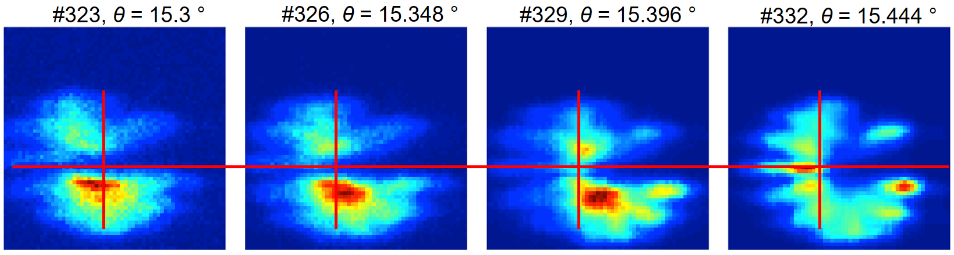

Figure S5. **Inversion of the 3D dataset using classical Bragg ptychography approach**. Amplitude (arbitrary units) and phase (radians) of the retrieved object, obtained using the classical Bragg ptychography approach based on the 3D Fourier transform formalism. No initial pre-alignment of the data (cross-correlation alignment) was performed. Convergence is obtained after 200 iterations.

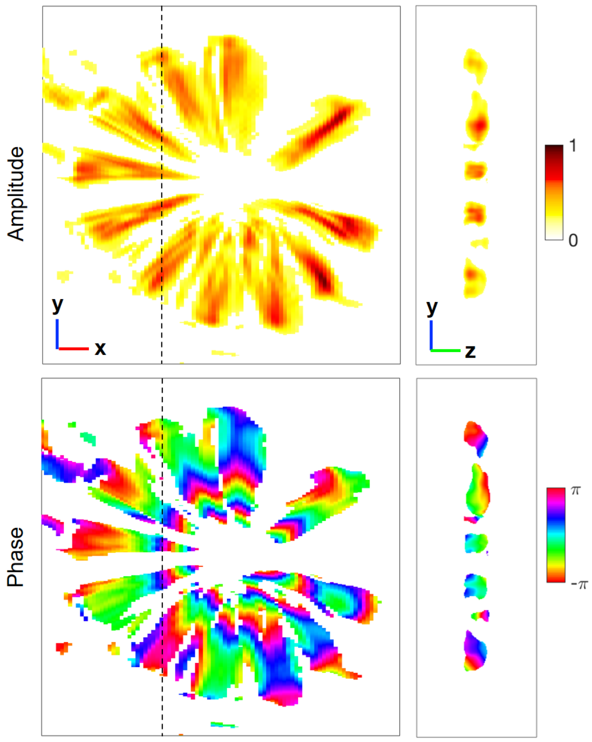

Figure S6. Inversion of the 3D dataset using classical Bragg ptychography approach and preliminary cross-correlation alignment of the data. Same as Fig. S5, introducing the preliminary alignment of each ptychography spatial scan (see Fig. S4), based on the cross-correlation analysis of two successive spatial scans. Convergence is obtained after 200 iterations. Results are slightly improved with respect to Fig. S5.

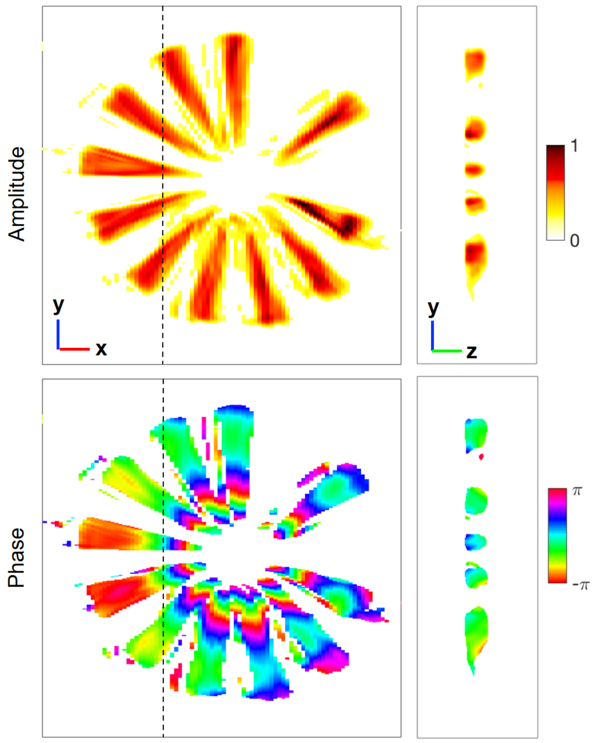

Figure S7. Inversion of the 3D dataset using the new approach. Amplitude (arbitrary units) and phase (radians) of the retrieved object, obtained using the approach described in the "Bragg ptychography inversion details" section above. The quality of the reconstruction is unambiguously good.

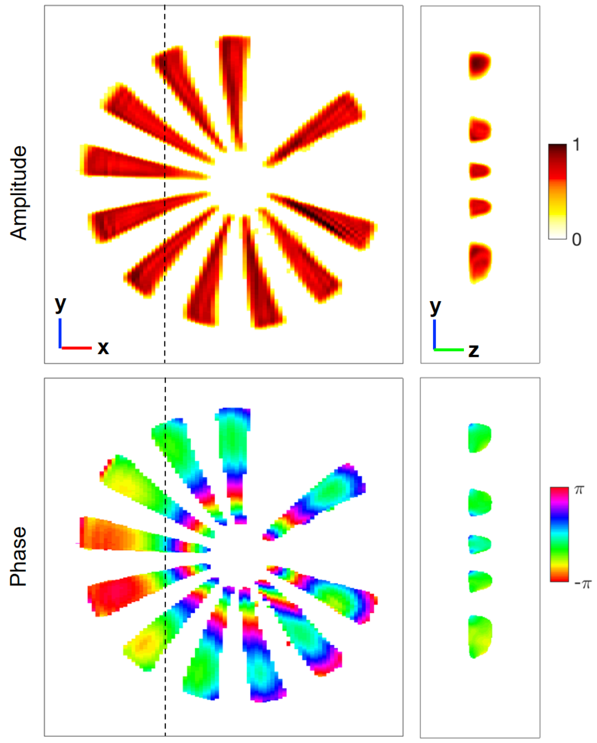

Figure S8. **Test of the 3D Bragg ptychography inversion process.** Amplitude (arbitrary units) and phase (radians) of the retrieved object. The numerical Bragg ptychography data were created using the 3D amplitude shown in Fig. S7. No strain was added (*i.e.*, only using constant phase) in order to evidence the phase fluctuations arising from the inversion. The scan parameters were chosen to mimic the experimental data parameters, including the fly-scan mode and the photon shot noise (Poisson statistics), corresponding to a maximum intensity per pixel of 300 photons. Furthermore, spatial drifts were added within each ptychography scans and in between two successive scans. These global ones were the same as the experimentally determined ones while the local drifts were random.

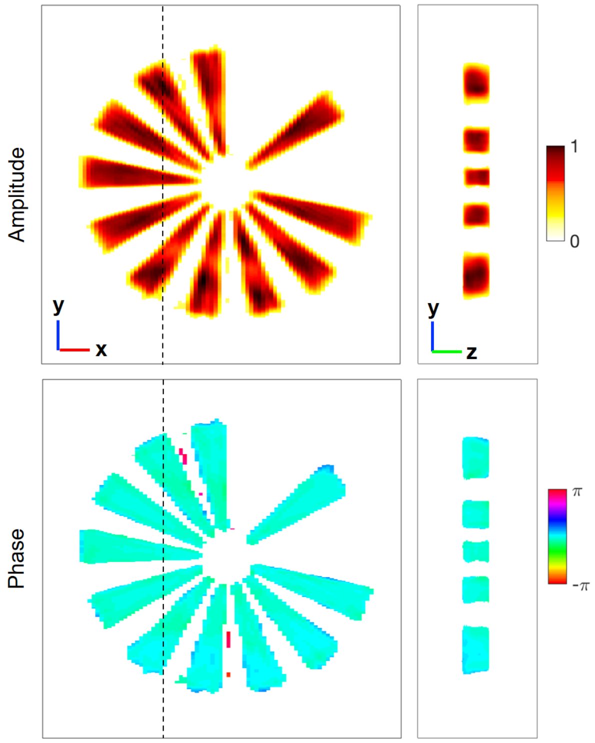

Figure S9. Test of the 3D Bragg ptychography inversion process. Same as Fig. S8, considering a step-scan mode for the ptychography scan.

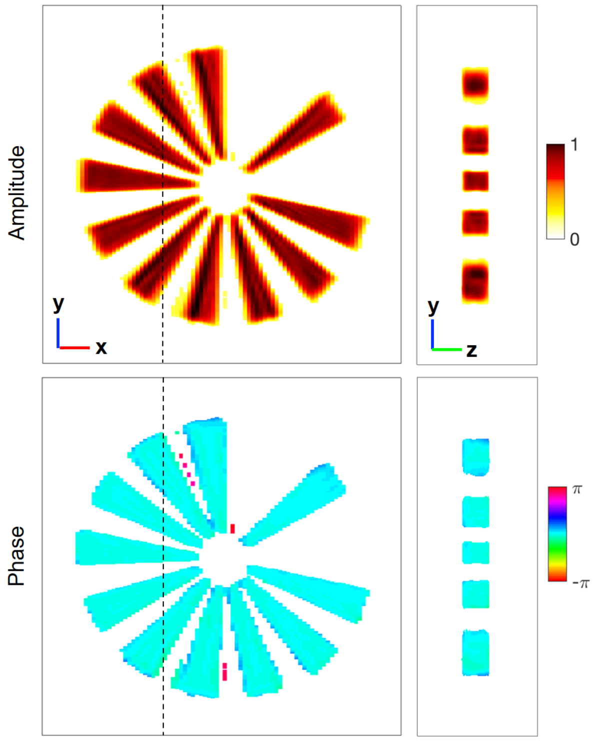

Figure S10. L-curves analysis. **a – b**, L-curve analysis tested on simulated data (no noise, no drift) for estimating the thickness and the tilt angle parameters, respectively. **c – d,** same as **a** and **b** for simulated data poised by Poisson noise (maximum of intensity fixed to 300 counts pixel^{-1}). **e – f,** L-curve analysis applied to the 3D Bragg ptychography data set.

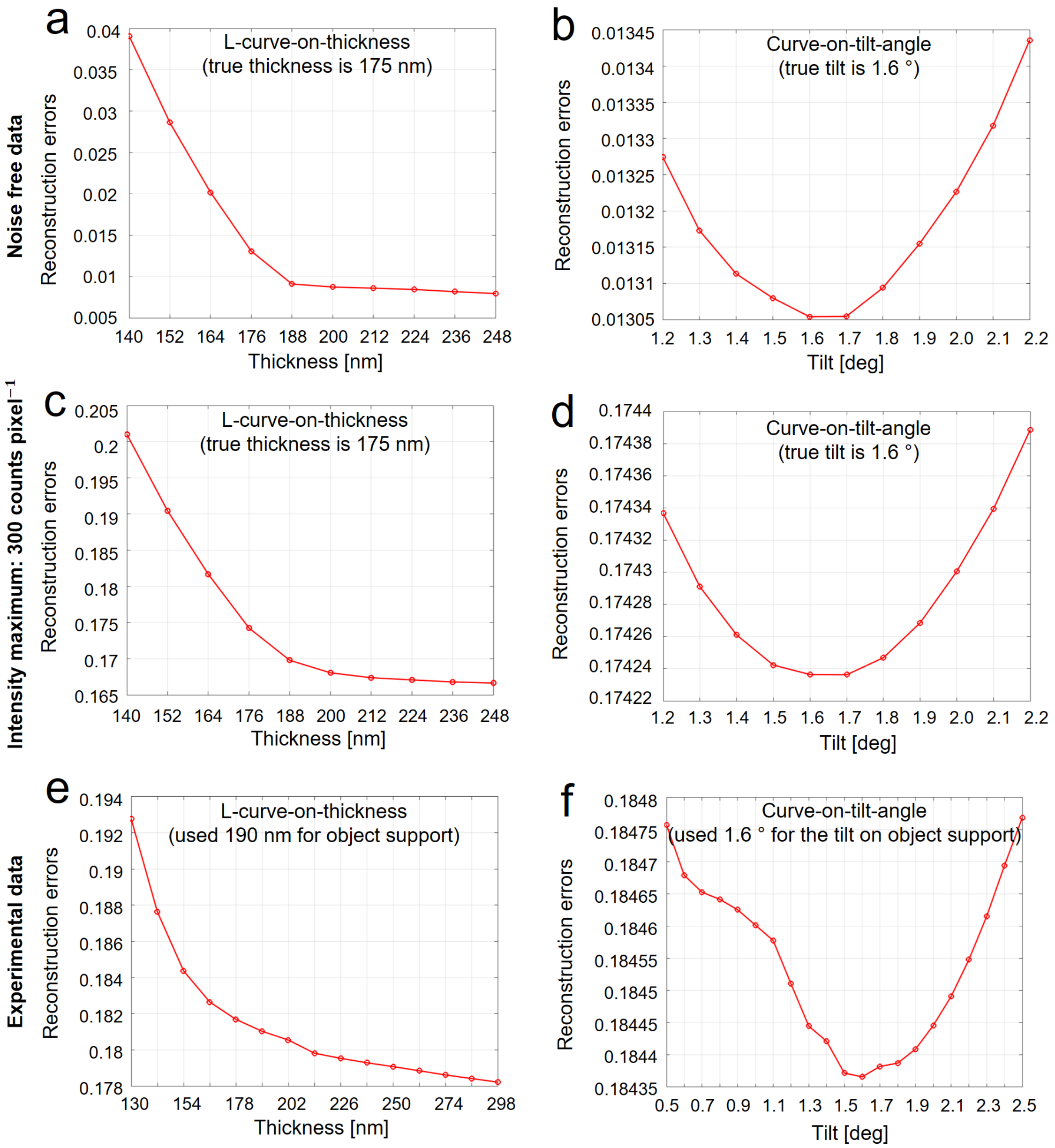

Figure S11. Inversion of the 3D data-set using the new approach (no threshold constraint). Same as Fig. S7, without using the threshold constraint during the inversion.

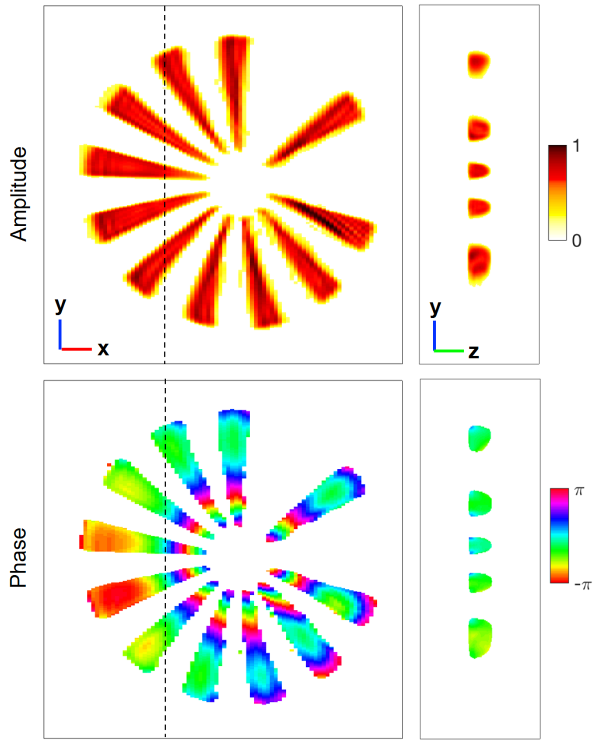

Figure S12. 3D spatial resolution estimation. Fourier shell correlation analysis calculated for the full Fourier components (3D averaged) and along the three **z**, **y** and **x** directions (of results shown in Fig. S7). The estimated resolution values are indicated on the plots in red.

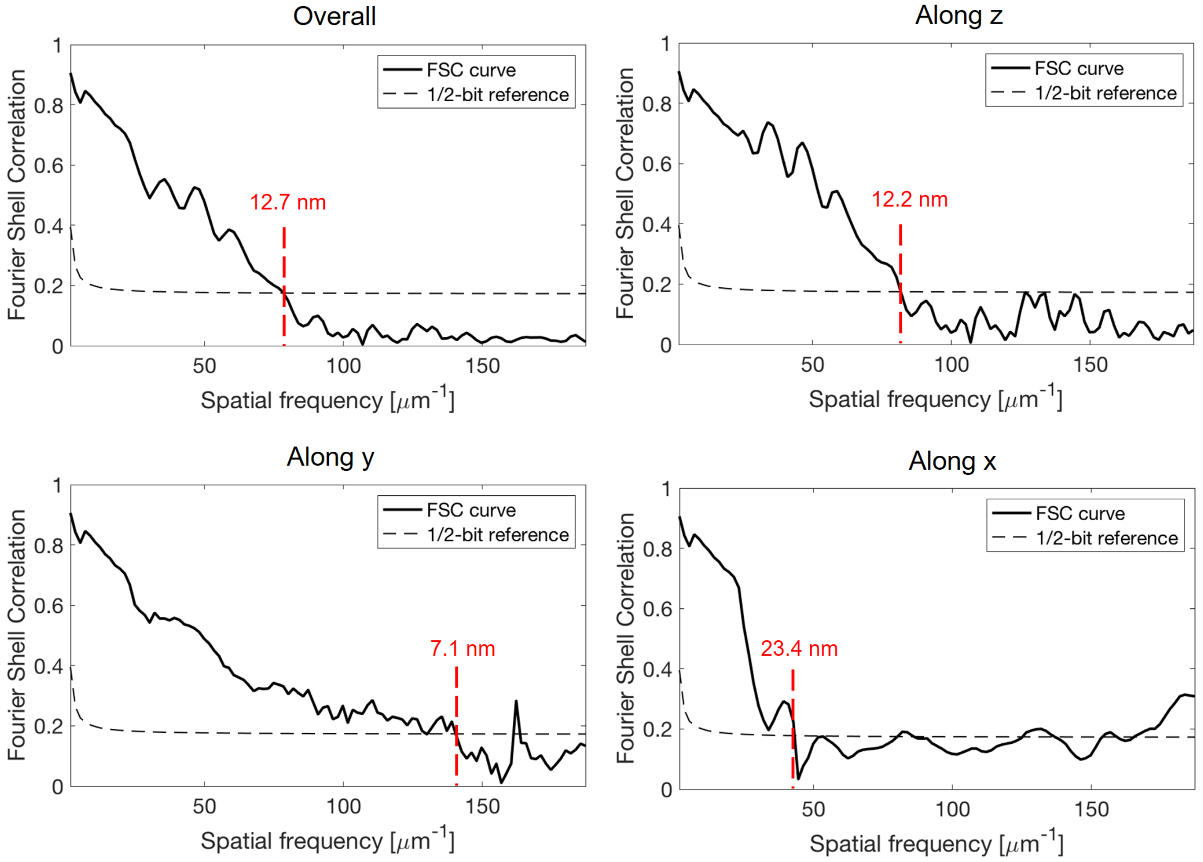

Figure S13. Impact of the photon shot noise on the retrieved probe mode power distribution. a, Relative probe mode power distribution retrieved from simulated data, introducing different photon shot noise levels, from noise free down to a maximum of photons of 3.10^1 photons. A clear change in the power mode distribution is visible, with more power in the first modes when the noise increases. **b – g,** 2D diffraction patterns at a fixed scan position, showing the degradation of the signal from the noise free case **b** to the less intense (i.e., noisier) case at 3.10^1 photons **g**. The maximum photon count is indicated on the plots.

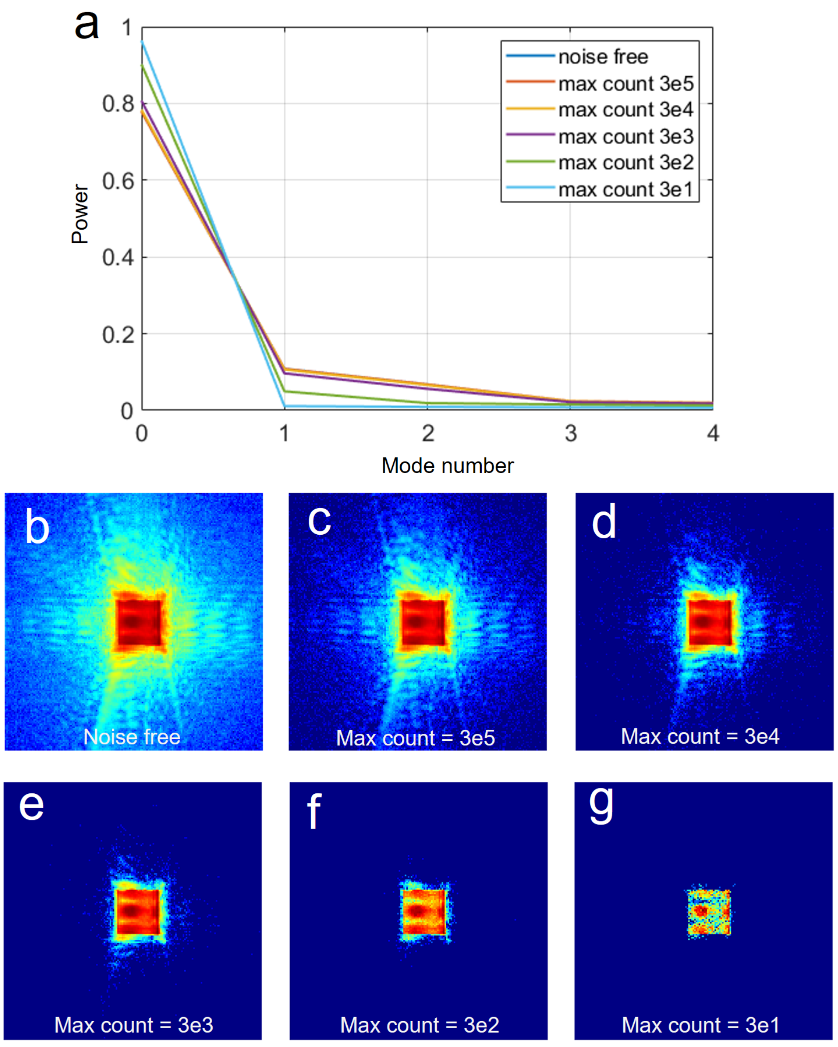

Figure S14. Retrieval of the global drifts and local drifts (exp. data). Inversion of the experimental data shown in Fig. S7. **a,** Retrieved values of the global drifts (along **x** and **y**) observed between two successive 2D ptychographical scans (*i.e.*, for a fixed angular position). **b** – **d,** Retrieved values of the local drifts, shown as red arrows with respect to their nominal values, for 3 different values of the θ angle (expressed as the difference with respect to θ_B). **e,** Zoom-in view corresponding to the region shown in **c** (red square). Note that the beam size (including tails) being much larger than the distance between two successive positions, the overlapping condition is always verified. Furthermore, the discrepancies between nominal and retrieved positions are different along the rocking curve, ensuring that the beam covers all parts of the sample in a homogeneous manner.

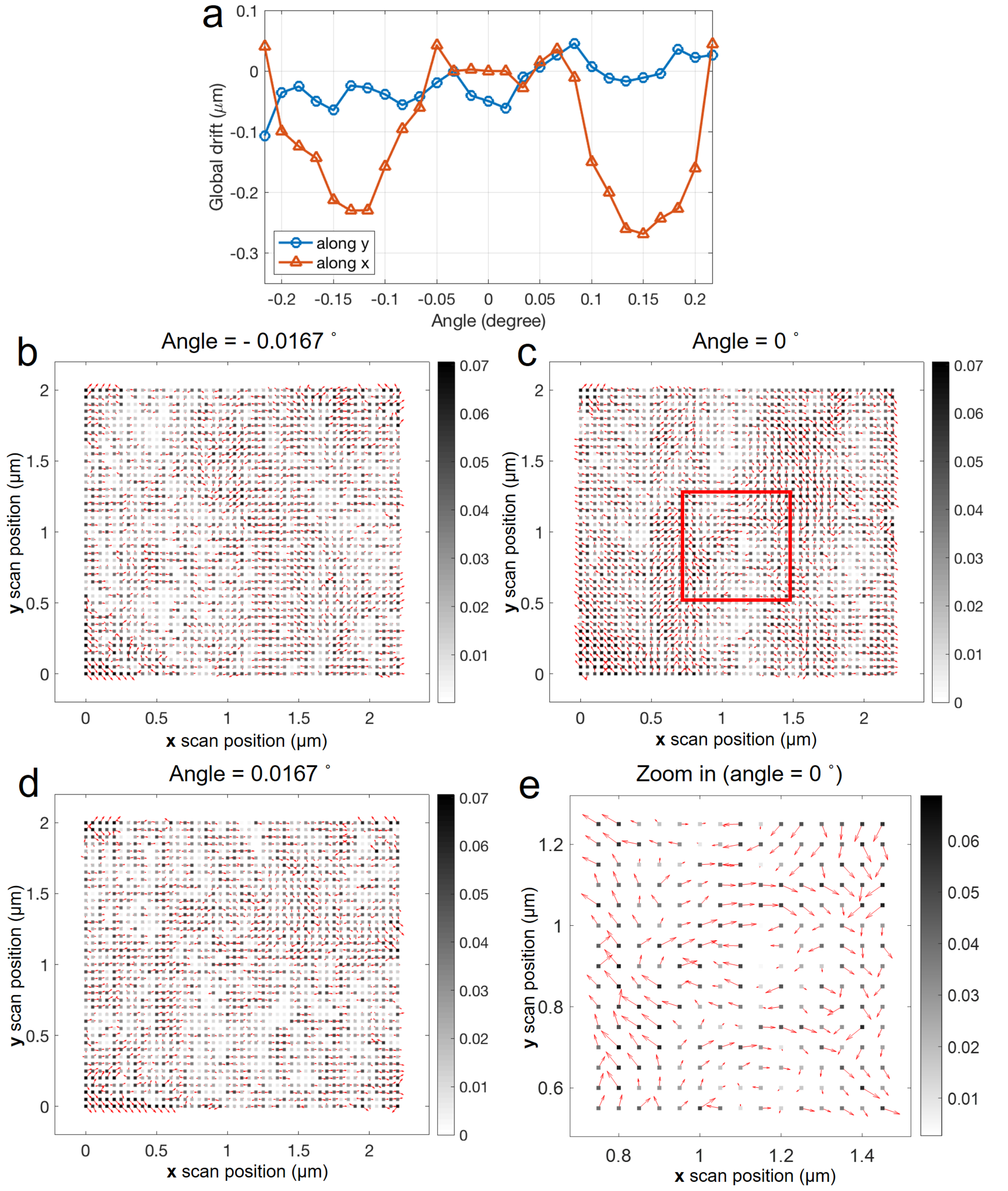

Figure S15. Test of the 3D Bragg ptychography inversion process - Reference reconstruction. Same as Fig. S8, including the fly-scan mode. However, no spatial drift was introduced and the intensity level was cropped to 300 photons without being poised by the Poisson noise.

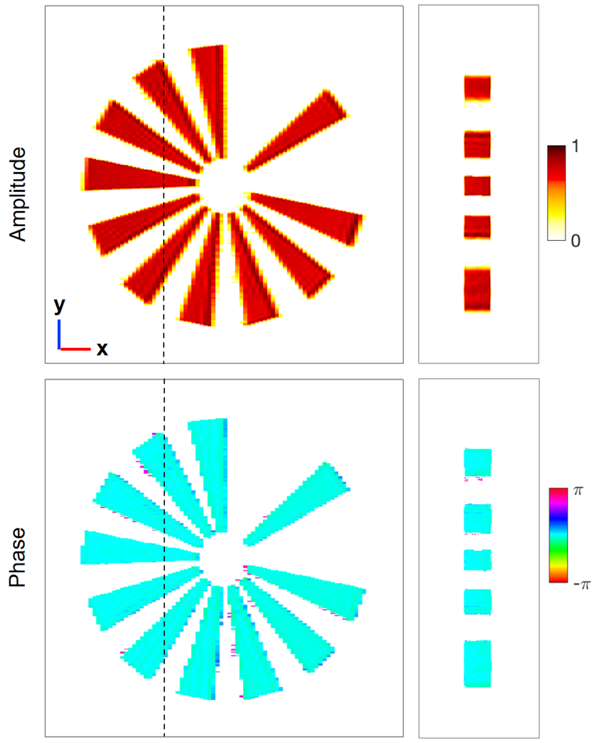

Figure S16. Test of the 3D Bragg ptychography inversion process - (Poisson noise). Same as Fig. S15. However, the intensity (limited to 300 photons per pixel) was further poised by the Poisson noise.

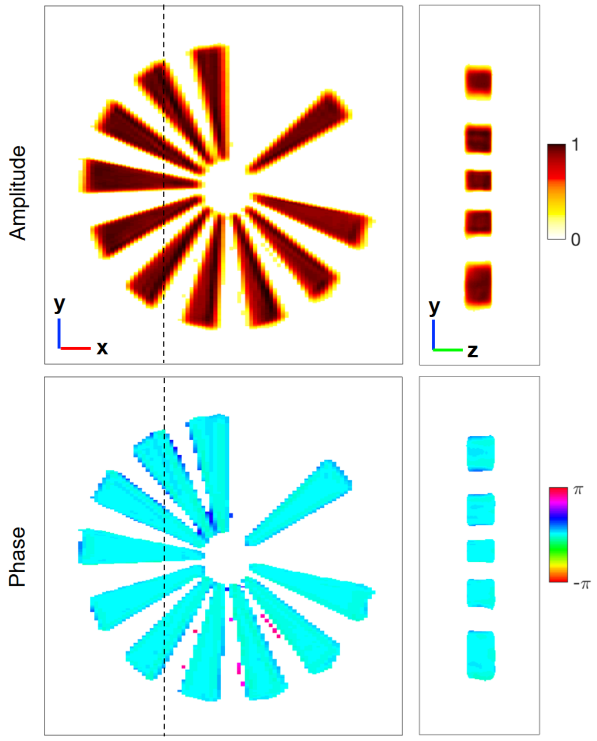

Figure S17. Test of the 3D Bragg ptychography inversion process - (step-scan). Same as Fig. S15. However a step-scan mode was considered to produce the ptychographical data set.

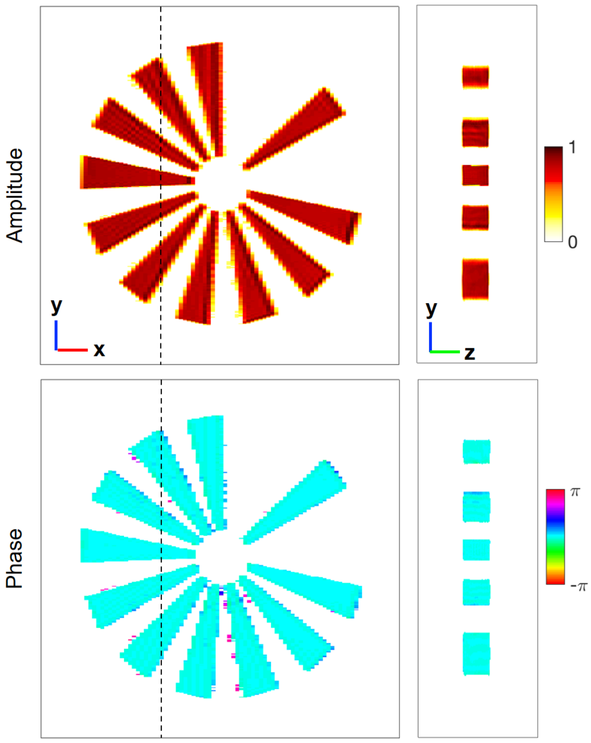

Figure S18. Investigation of the minimum number of angular steps along the rocking curve. Same as Fig. S7, using only one half of the angular information along the rocking curve (*i.e.*, corresponding to a double angular step size).

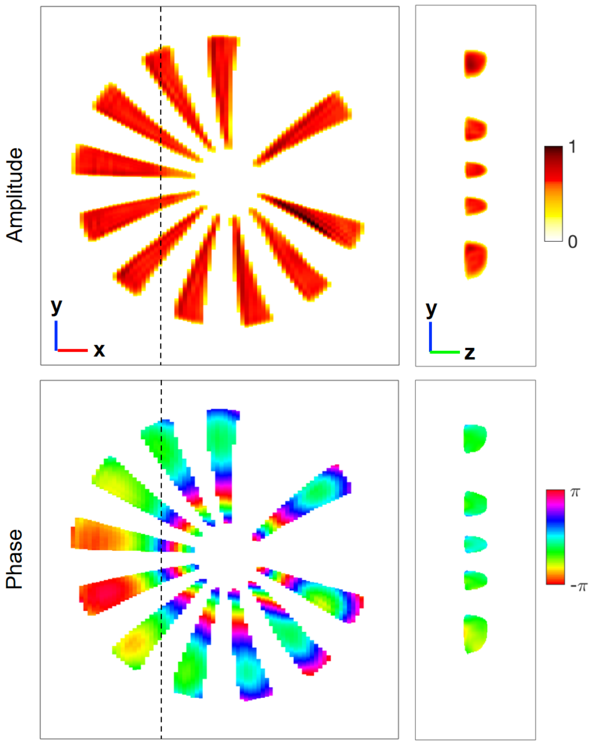

Figure S19. **Investigation of the minimum number of angular steps along the rocking curve.** Same as Fig. S7, using only one third of the angular information along the rocking curve (*i.e.*, corresponding to a triple angular step size). The retrieved image is slightly degraded.

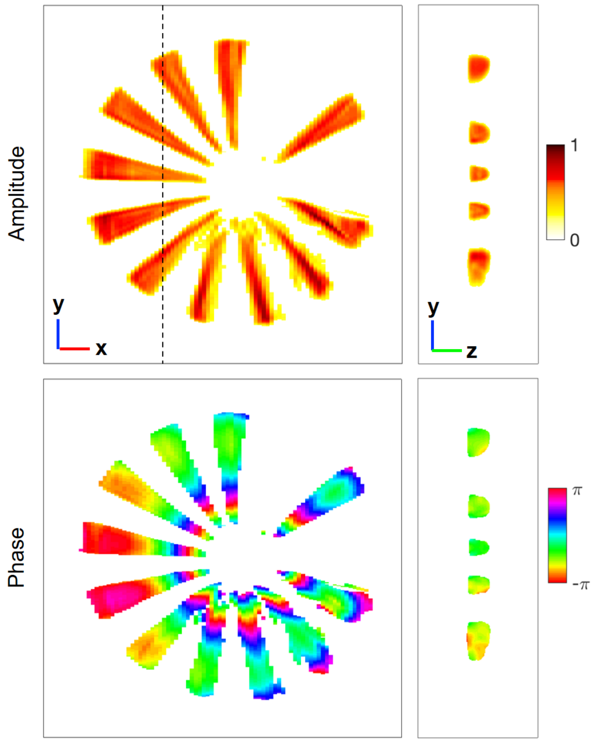

Table S1. Comparison with other result. Summary of the main experimental characteristics and retrieved image properties for reference (1) and this work. These include the coherent flux at sample position *F*, the retrieved field of view *FOV*, the 3D spatial resolution, the acquisition time *t*, the sample volume *V* and the integrated intensity measured at the detector planes *I*. The last column presents the respective gains defined as the ratio between this result and reference (1), except for *t* and the resolution, for which the gain is defined as the ratio between reference (1) and this result. Note that the low coherent flux in reference (1) results from a strong selection of the beam prior and the use of relatively less efficient Fresnel zone plate to produce the focal spot.

	**Reference** (1)	**This result** (Fig. S7)	**Gain** (see caption)
F (ph s^{-1})	10^8	$6 \cdot 10^9$	60
FOV (μm^2)	0.86	13.31	16
3D resolution (nm^3)	50 × 45 × 15	7 × 12 × 23	17
t (s)	12600	1644	8
V (μm^3)	0.073	0.208	3
I (photons)	$3.37 \cdot 10^7$	$3.31 \cdot 10^7$	1

(1) V. Chamard, M. Allain, P. Godard, A. Talneau, G. Patriarche, M. Burghammer, Strain in a silicon-on-insulator nanostructure revealed by 3D x-ray Bragg ptychography. *Sci. Rep.* **5**, 9827 (2015).
